# Cannabis and Canabidinoids on the Inflammatory Bowel Diseases: Going Beyond Misuse

**DOI:** 10.3390/ijms21082940

**Published:** 2020-04-22

**Authors:** Antonelly Cassio Alves de Carvalho, Gabriela Achete de Souza, Samylla Vaz de Marqui, Élen Landgraf Guiguer, Adriano Cressoni Araújo, Claudio José Rubira, Ricardo de Alvares Goulart, Uri Adrian Prync Flato, Patricia Cincotto dos Santos Bueno, Rogério Leone Buchaim, Sandra M. Barbalho

**Affiliations:** 1 Postgraduate Program in Structural and Functional Interactions in Rehabilitation, University of Marilia (UNIMAR), Avenue Hygino Muzzy Filho, 1001, Marília 17525902, São Paulo, Brazil; drantonelycarvalho@hotmail.com (A.C.A.d.C.); elguiguer@gmail.com (É.L.G.); adrianocressoniaraujo@yahoo.com.br (A.C.A.); ricardogoulartmed@hotmail.com (R.d.A.G.); uriflato@gmail.com (U.A.P.F.); rogerio@fob.usp.br (R.L.B.); 2 School of Medicine, University of Marília (UNIMAR), Avenida Higino Muzzi Filho, 1001, Marília 17525902, São Paulo, Brazil; gabriela.achete@outlook.com (G.A.d.S.); Samyllavaz@outlook.com (S.V.d.M.); claudio.rubira@gmail.com (C.J.R.); pcincotto@gmail.com (P.C.d.S.B.); 3 Department of Biochemistry and Nutrition, Faculty of Food Technology of Marília, Marília 17525902, São Paulo, Brazil; 4 Department of Animal Sciences, School of Veterinary Medicine, University of Marília (UNIMAR), Avenida Higino Muzzi Filho, 1001, Marília 17525902, São Paulo, Brazil; 5 Bauru School of Dentistry, Department of Biological Sciences, University of São Paulo (FOB–USP), Alameda Doutor Octávio Pinheiro Brisolla, 9-75, Bauru 17040, São Paulo, Brazil

**Keywords:** inflammatory bowel disease, ulcerative colitis, Crohn’s disease, cannabis, cannabidiol

## Abstract

Inflammatory bowel diseases (IBD) are characterized by a chronic and recurrent gastrointestinal condition, including mainly ulcerative colitis (UC) and Crohn’s disease (CD). *Cannabis sativa* (CS) is widely used for medicinal, recreational, and religious purposes. The most studied compound of CS is tetrahydrocannabinol (THC) and cannabidiol (CBD). Besides many relevant therapeutic roles such as anti-inflammatory and antioxidant properties, there is still much controversy about the consumption of this plant since the misuse can lead to serious health problems. Because of these reasons, the aim of this review is to investigate the effects of CS on the treatment of UC and CD. The literature search was performed in PubMed/Medline, PMC, EMBASE, and Cochrane databases. The use of CS leads to the improvement of UC and CD scores and quality of life. The medical use of CS is on the rise. Although the literature shows relevant antioxidant and anti-inflammatory effects that could improve UC and CD scores, it is still not possible to establish a treatment criterion since the studies have no standardization regarding the variety and part of the plant that is used, route of administration and doses. Therefore, we suggest caution in the use of CS in the therapeutic approach of IBD until clinical trials with standardization and a relevant number of patients are performed.

## 1. Introduction

Inflammatory bowel diseases (IBD) are characterized by a chronic and recurrent gastrointestinal condition that can result from complex interactions between genetic and environmental factors leading to intestinal inflammation. They can affect 200 people for every 100,000 adults in the United States and 400 for every 100,000 in the United Kingdom. Ulcerative colitis (UC) and Crohn’s disease (CD) are the primary forms of IBD, and the growing incidence is associated with genetic factors, stress, a diet rich in sugars and fats, reduced fiber and vitamin intake, and consumption of xenobiotics. The primary symptoms related by the patients include abdominal pain, diarrhea, rectal bleeding, and weight loss [1,2,3,4]

There is still no effective management and treatment for these recurrent chronic diseases. The available therapies are usually associated with numerous side effects and represent a high cost for patients. The medications commonly used are corticosteroids, agents derived from acetylsalicylic acid, anti-TNFα, and other immunomodulatory drugs. Although these drugs are useful in many patients, others do not benefit from the results of these therapies. Also, they can lead to opportunistic infections, bone marrow suppression, malignancy secondary to immunosuppression, and reactions caused by the medication infusion procedure [5]. As a result, unconventional treatments can assist in maintaining or inducing remission and are usually inexpensive. One possibility of therapeutic adjuvant that has been explored by physicians is the use of *Cannabis sativa* (CS) or its derivatives [4,6].

CS is a subspecies of the genus Cannabis and has been used for therapeutic or recreational purposes since ancient times. The pioneers in this use were possibly the Chinese and Indians. It is characterized by containing aromatic hydrocarbons called cannabinoids and terpenes, mainly located in the trachoma cavity of the plant. These compounds seem to play a substantial role as antioxidant and anti-inflammatory properties and can be beneficial in the therapeutic approach of several diseases such as Alzheimer’s disease, Parkinson’s disease, neuropathic pain, chronic pain, anxiety, schizophrenia, and cancer. IBD patients can also benefit from the use of this plant or its derivatives [7,8,9,10,11,12,13].

Besides that, there is still much controversy about the use of CS since the misuse of this plant can lead to serious health problems. On the other hand, the literature shows that it can be useful in the treatment of IBD patients that are refractory to conventional therapies [14]. Because of these reasons, this review aims to investigate the effects of CS on the treatment of UC and CD.

## 2. Results

**Figure 1:**
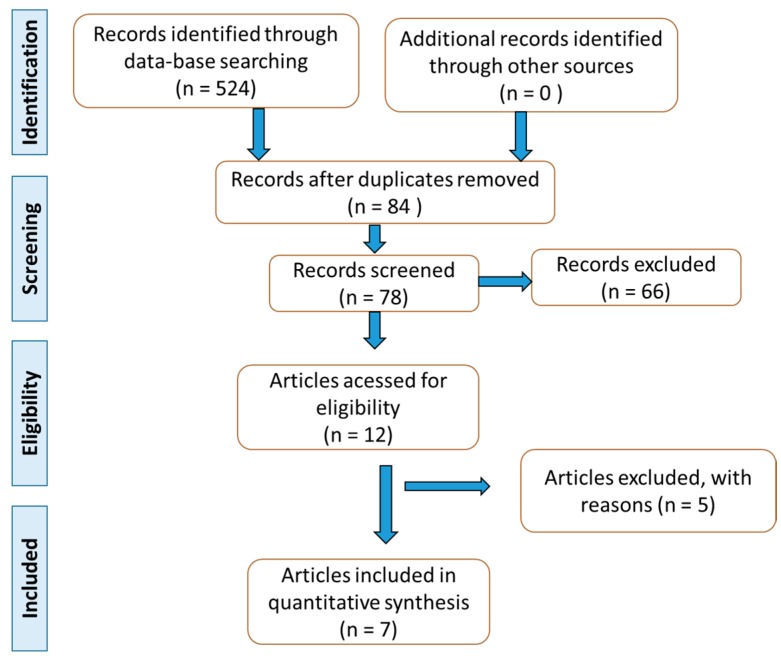
Flow diagram according to PRISMA guidelines [15].

Based on the list of the selected studies (Figure 1), the authors build Table 1 Other studies with animal models and in vitro studies were used to build the discussion.

The articles selected for this review included one retrospective observational study [16], one prospective observational study [17], two prospective placebo-controlled trial [18,19], one longitudinal, Internet-based cohort study [20], one double-blind placebo-controlled, parallel-group study [21], and one population-based cohort study [22]. Five studies were performed in Israel, one in the United Kingdom, and one in the United States.

One hundred thirteen individuals were involved in five RCT (62 with UC and 51 with CD), 39,802 were included (298 were CS users) in a population-based cohort study, 1166 individuals (1045 with CD and 121 with UC) were involved in a longitudinal, Internet-based cohort study, and 30 individuals were included in a retrospective observational study.

## 3. Discussion

### 3.1. Inflammatory Bowel Diseases

Despite a sharp increase of patients with IBD, UC is more prevalent than CD and shows a continuous pattern of inflammation that is restricted to the surface of the colonic mucosa. The extent of inflammation is uniform and rarely affects the terminal ileum. It can affect all ages, but peaks of incidence are usually found between the third and fourth decade of life. The patient often presents tenesmus, blood in the stool, weight loss, diarrhea, flatulence, and abdominal pain, resulting in a substantial decrease in quality of life. Furthermore, it is related to psychological disorders [23,24].

The etiological variables associated with the development of UC are still not completely understood. Some researchers have shown that an excessive pattern of immune response together with psychological factors, diet, and genetic predisposition may be crucial for the development of this condition [25,26].

**Figure 2:**
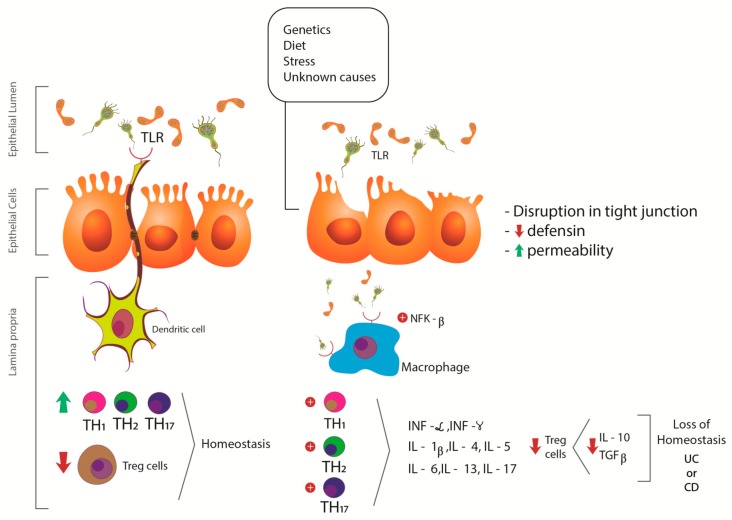
Pathophysiologic aspects of UC and CD. Genetic and environmental factors may be related to the disruption in tight junctions and the increase in permeability, leading to an abnormal immune response. The consequences are imbalanced stimulation of TLR and NFκ-β, leading to overexpression of pro-inflammatory biomarkers (IL-1β, IL-4-6, IL-17, TNF-α, and INF) and reduced expression of IL-10 and TGF-β. TLR: Toll like receptor; NFk-β: nuclear factor kappa-beta; INF-γ: interferon-γ; TNF-α: tumor necrosis factor- α; IL: interleukin; TH: T helper cell; Treg cells: T regulatory cells; UC: ulcerative colitis; CD: Crohn’s disease.

CD consists of a segmental granulomatous inflammation that mainly affects the terminal ileum and colon, and can reach from the oral cavity to the anus and differs from the UC, mainly because of the “skipped” pattern of the inflammation. In the relapse phase of the disease, the patient has abdominal pain, diarrhea, bleeding, fever, and weight loss. As in UC, psychological factors may be associated. Other complications that can be observed in patients are strictures, fistulas, and abscesses. The pathophysiology of CD is also not wholly elucidated, but microbial exposure, genetic, and environmental factors may be associated [27,28]. Figure 2 shows some pathophysiologic aspects of UC and CD.

There are about 200 cases of CD for every 100,000 inhabitants in North America and 100 for every 100,000 inhabitants in Western Europe. In the city of São Paulo, there are an estimated 14.8 cases for every 100,000 inhabitants. About 25% of the diagnoses occur before 18 years, but the peak of the disease occurs between 20 and 40 years of age. About 75% of patients need surgical interventions to correct complications resulting from the disease. As in the UC, patients with CD may also experience periods of remission and periods of crises [29,30,31].

The increasing rates of patients with UC or CD have increased the search for therapies to patients that are refractory to conventional therapy, and that exhibit fewer side effects. For these reasons, medicinal plants have been gaining the attention of researchers worldwide.

### 3.2. Cannabis sativa and Endocannabinoid System

CS is one of the most widely consumed plants worldwide and exhibits a long history in medicinal preparations, recreational use, and use in religious rituals. The leaves can be consumed orally or by inhaled vapors, and the seeds can be used in the production of cooking oils. CS has about 100 active phytochemicals known as cannabinoids. Among them, the most studied is Δ9-tetrahydrocannabinol (THC) and cannabidiol (CBD) that can activate endogenous Cannabinoid Receptors 1 (CB1) and 2 (CB2). Cannabigerol (CBG), cannibro-chromene (CBC), and more than 100 other types of compounds are also present [32]. CS also presents a variety of terpenes (over 200 were described), including monoterpenes (linalool, limonene, and α-pinene) and sesquiterpenes (βcaryophyllene and (−)-α-bisabolol). α-pinene and limonene are some of the most common [7]. Table 2 shows some relevant phycompounds found in CS and general effects.

The endocannabinoid system (ECS) is composed of numerous endogenous receptors and ligands involved in several processes that can include hunger, perception of pain, gastrointestinal motility, and immune response. The targets of the ECS are the classic cannabinoid receptors (CB1 and CB2), as well as peroxisome proliferator-activated receptors (PPARs), the orphan GPR55 receptor, and TRPV (transient potential vanilloid receptor). These targets are found in the gastric mucosa, the enteric nervous system, and the immune system. Endocannabinoids, such as anandamide and 2-arachiodioylglycerol are molecules related to intercellular lipid signaling resulting from the demand for membrane precursors [32,33,34].

Scientists found a place for THC and CBD in clinical practice in the treatment of different pathologies such as arthritis, muscle spasticity observed in multiple sclerosis, chronic pain, and in childhood epilepsy. The literature shows several synthetic cannabinoid agonists that keep growing. These compounds exhibit a high selective affinity for CB1, CB2, GPR55, and TRPV1 receptors and have been evaluated pre-clinically as having effects on immunity and intestinal motility [35,36,37].

During the inflammatory process, there is an increase in the expression of CB1, CB2, PPARα, and PPARγ receptors in the submucosa and the adjacent immune cells, while the expression of GPR55 and TRPV1 is reduced in the mucosa and increased in the enteric nervous tissue [38].

Studies with animal models of colitis have shown that the use of cannabinoids prevents the onset of experimental colitis or minimizes its severity. Clinical studies have also investigated the effects of cannabinoid ligands or the effect of blocking their metabolizing enzymes on inflammation of the intestine and have shown significant promising preclinical evidence for the treatment of colitis [39,40].

In vitro studies show that the anti-inflammatory property of CS extracts on colon epithelial cells is due to a fraction of the extract that contains THCA, mediated at least in part, by GPR55 receptor [41]. Couch et al. [42] evaluated the effects of cannabidiol and palmitoylethanolamide in cultured cell lines and in inflamed explant human colonic tissue and found that the treatment with tumor necrosis factor-α (TNF-α) and interferon-γ (IFN-γ) augmented cytokine and phosphoprotein levels in Caco-2 cultures and colonic explant. The use of both cannabinoid drugs reduced phosphoprotein levels in Caco-2 cultures and colonic explants. Cannabidiol and palmitoylethanolamide prevented an increase in cytokine production in explant colon and showed anti-inflammatory actions in the human colon.

Beyond the psychogenic effects of THC, it also exhibits antioxidant, anti-inflammatory, and analgesic properties. The psychoactive effect occurs because of the agonist action on CB1 receptors, located mainly in the brain, in addition to promoting the mediation of neuronal inhibition by decreasing the calcium influx and increasing the potassium efflux through the cell membrane. CB1 receptors are found in inhibitory (GABA-ergic) and excitatory (glutamatergic) neurons. THC is also a partial CB2 agonist, which is mainly distributed in immune and hematopoietic cells [43].

CBD is one of the main non-psychoactive phytocannabinoids present in CS, representing 40% of the extracts of this plant. Its effects are 5-HT1a, α3, and α1 glycine receptor agonists and exhibit weak binding with CB1 receptors. It has anti-inflammatory, anti-apoptotic, neuroprotective, and modulating effects on intracellular Ca^2+^ concentration [44].

**Figure 3:**
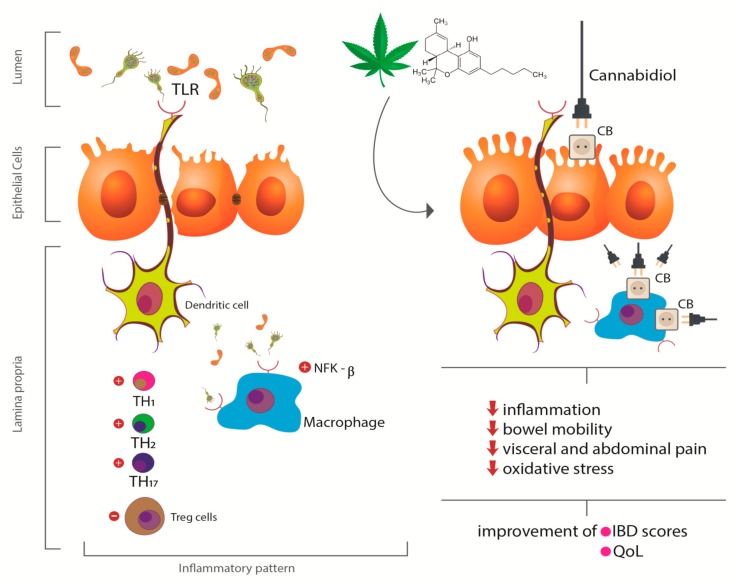
Effects of Cannabis sativa or its derivatives in the pathophysiologic aspects of IBD. When acting on peripheral CB receptors, canabidiol decreases the inflammatory response by decreasing TLR and NFK-β activation, reduces the generation of free radicals and oxidative stress, and reduces intestinal motility leading to consequent improvement in quality of life. TLR: Toll like receptor; CB: canabidiol receptor; NFk-β: nuclear factor kappa-beta; TH: T helper cell; Treg: T regulatory cells; IBD: inflammatory bowel diseases; QoL: quality of life.

Studies have shown that agonists of cannabinoid receptors exert inhibitory properties of small intestine peristalsis through the activation of CB1 receptors. This process suppresses propulsive peristalsis and contractions of the ascending enteric reflex inhibiting cholinergic and non-cholinergic transmission. CB1 agonists can reduce the relaxation of the lower esophageal sphincter, leading to reduced gastroesophageal reflux. CB2 agonists are related to the decrease of oxygen free radicals by the intestinal epithelium. In the inflamed mucosa, activation of CB1 and CB2 receptors can reduce the hypermotility associated with inflammation. Cannabinoids also exert analgesic, anti-nausea, and anti-diarrheal effects in patients with IBD [43,45,46,47]. Figure 3 shows the effects of CS or its derivatives in the pathophysiologic aspects of IBD.

Finally, it is important to consider that CS has already been described as a very rich source of bioactive compounds and, despite producing more than 100 cannabinoids, the focus has been on THC and CBD. However, numerous secondary metabolites, especially terpenes, share the common intermediate geranyl diphosphate with cannabinoids, which may contribute synergistically to the beneficial effects of the plant and its compounds [48]. This effect, called entourage (initially described by Ben-Shabat et al. [49]), is a type of synergism that would explain the hypothesis that in some cases, the plant would be better than the isolated compounds. Thus, part of the beneficial effects would be due to a combination of cannabinoids and terpenes and, in this sense, some studies have shown that for some diseases the Cannabis extract would have a potency four times greater than THC alone [50].

It is also worth remembering that the use of CS still leads to numerous questions about effectiveness and safety, making healthcare professionals still very concerned about the adverse effects such as psychotropic side effects, increase food intake, short-term memory, and other cognitive deficits [13,51].

### 3.3. Inflammatory Bowel Diseases and Cannabis sativa

Authors have shown that IBD patients commonly use complementary and alternative therapies to alleviate symptoms of IBD. Patients report that the use of CS is associated with the improvement of abdominal pain, mood, and sleep [52,53].

In a survey with IBD patients of a tertiary care IBD clinic in Canada, Hansen et al. [4] surveyed individuals regarding the use of CS, mental health symptoms, and personality risk factors related to substance misuse. From the 201 patients that answered the questionnaire, 108 reported cannabis use, most of them were CD patients. The authors also found that the use of CS was more likely to be used by patients with moderate-severe depressive symptoms and smokers.

Jamal et al. [54] performed a retrospective chart review with patients undergoing elective IBD surgery to compare the use of opioids in the first twenty-four hours post-surgery between patients who reported pre-surgery cannabis use and those who did not report. A total of 354 individuals was included in the study, and 88.1% were cannabis nonusers, and 11.9% were users. The authors found that patients consuming cannabis before the surgery show higher opioid need in the post-operative stage.

Kerlin 2018 [20] study included 1666 participants with IBD (CD = 1045 and UC = 121) who completed a health survey and provided updates after six months. These participants were divided between patients who used cannabis, whether it was prescribed or recreational, and patients who did not use cannabis. Participants who used CS reported clinical improvement, but also other symptoms such as increased anxiety and increased depression. The application of this questionnaire may result in bias, considering that the inquiry pointed out two distinct diseases and a great group of individuals who also do not have standardized use. It is worth mentioning that patients in more severe stages of the disease used prescribed cannabis because they did not respond adequately to the conventional treatments, and this can generate differences between users who did not present such severe symptoms.

The Lahat et al. 2012 [17] study investigated 13 patients diagnosed with IBD that used three inhalations of cigarettes of dry cannabis when they were in pain. In addition to the small number of participants, the authors did not report if the disease was active or not. Moreover, the study does not present a detailed composition of the product that was used. There was also no standardization in the doses used (the indication was three inhalations in situations of pain). There is no specification of the evolution individually, considering that they treat two different diseases. Despite the biases presented, positive results were noted in the use of CS for the treatment of IBD, seen by the decrease in pain, improvement in the general perception of health, improvement in the patients’ workability, and weight gain.

The summary for the studies involving IBD and CS and those that will be discussed below can be found in Table 1.

### 3.4. Ulcerative colitis and Cannabis sativa

Only two studies investigating the effects of CS in UC patients were found in the databases that were consulted. Irving et al. [21] evaluated UC patients that received a regular oral dose of CBD-rich botanical extract that reached 250 mg, twice, for eight weeks. Even with a lower tolerance to CBD-RBE, the treated group reported an improvement in the quality of life compared to placebo, but there was no statistical relevance regarding clinical improvement. The study was limited by low tolerability, even having a larger sample compared to other experimental studies. For these reasons, further studies should be performed to verify a more appropriate dosage and route of administration with the most significant benefit.

In a population-based cohort study, Mbachi et al. [22] showed that the use of CS for UC patients is associated with reduced prevalence of bowel obstipation and hospital length-of-stay when compared with nonusers. The authors postulate that the plant can mitigate some complications of UC.

### 3.5. Crohn´s Disease and Cannabis sativa

As in UC, not many studies that investigated the use of CS and CD are found in the literature. Naftali et al. 2011 [16] interviewed 30 patients with CD. Although this was a pioneering study in showing the use of CS in IBD, there was no comparison with placebo, the presence of adverse effects was not investigated.

In the RCT of Naftali et al. 2013 [18], the effects of CS in CD patients were investigated. However, it is difficult to maintain the blindness of the study, as psychotropic effects are noted in the group that received the intervention, even if the recruited patients were laymen to the compound, most patients were able to report which group they were allocated. Studies that use oral use of CS may have this complication reduced. Furthermore, the number of patients studied was not large, and there was no significant clinical improvement compared to placebo. Perhaps in a larger sample, other results will be obtained.

Another study by Naftali et at. 2017 [19] evaluated patients with CD that were treated with CBD. Within the group that received the treatment, there were six smokers, and this may have contributed not to generate statistical significance between treated and placebo groups since smoking worsens the prognosis of the disease. Studies also show that the combination of cannabinoids is more effective than the use of an isolated cannabinoid. In this study, the dose contained only one cannabinoid, avoiding synergism. Also, it is known that the use of cannabis through cigarette inhalation results in more significant effects compared to its oral use. Besides, the study involved a small number of participants, and this fact may compromise the statistical results.

### 3.6. Cannabis sativa, Inflammatory Bowel Diseases, and Adverse Effects

Three of the studies that investigated the use of CS on IBD patients showed similar side effects such as sleepiness, headache, nausea, and dizziness [18,19,21]. Three of them did not report the occurrence of adverse symptoms [16,17,22].

### 3.7. The Use of Cannabis sativa per se

The medical use of CS has been followed by controversy, especially concerning the safety of its isolated compounds. THC may cause undesirable effects such as conjunctival irritation, dysphoria, changes in spatial and temporal perceptions, anxiety, tachycardia, and also addiction (in higher doses) [55].

Unlike THC, there is little evidence that CBD alone produces THC-like psychotropic effects. The World Health Organization considered it to be well-tolerated and with good safety profile. In this sense, its anti-inflammatory effects can be useful and should be investigated in patients with IBD and other inflammatory conditions [56].

### 3.8. Final Comments

It is no longer possible to ignore the medicinal effects of CS, but many tortuous paths are yet to be followed. Studies have not shown a pattern in the variety and part of the plant that is used, in the route of administration (oral or inhaled), in the dose, and in the clinical moment of IBD. Indeed, knowledge about the effects of CS and its derivatives, how these compounds behave, and which is the best route of administration has not yet been exhausted. Only after the design of large, well-designed randomized controlled trials, it will be possible to finally discover the real benefits of this plant [10,57].

Furthermore, THC and CBD have received primary attention on the therapeutic effects of CS. However, this plant has other compounds such as terpenoids and flavonoids [7,58], which are known to have potent anti-inflammatory and radical scavenging potential. For these reasons, these molecules may also influence the effects of CS in IBD patients.

Another relevant bias is the small number of patients included in the studies, and the lack of side effects report in some of them.

## 4. Material and Methods

### 4.1. Search Strategy

The literature search was performed for observational, retrospective, or randomized controlled trials (RCTs) published in English from January 2010 to January 2020. The following combination of MeSH terms was used: Ulcerative Colitis or Crohn´s Disease or Inflammatory Bowel Diseases or colitis and Cannabis sativa or cannabinoids or THC or cannabidiol. The databases that were consulted were PubMed/Medline, PMC, EMBASE, and Cochrane.

### 4.2. Focal Question

The focal question used for this review was, “Is *Cannabis sativa* or cannabinoids effective in treating patients with IBD?”

### 4.3. Eligibility criteria and PICO (Population, Intervention, Comparison, and Outcomes)

The eligibility criteria for this search followed the *PICO* format for RCT. The studies involving patients with UC and CD who were treated or that regularly used CS or cannabinoids were included. Population-based cohort and longitudinal, Internet-based cohort studies were also included. Only full articles published in the consulted databases were selected.

### 4.4. Data Extraction and Selection of the Studies

Two independent reviewers (ACAC and SMB) in order to identify the researches in the databases performed the search for the studies involving the focal question. The abstracts of the studies were evaluated, and only full-text articles were retrieved to give support to the decision-making process. Disagreements between the reviewers were evaluated, discussed, and resolved by two other reviewers (ELG and ACA).

Inclusion criteria were observational, randomized clinical trials, cohort studies, cross-sectional studies, and case-control studies. The exclusion criteria included non-English articles, poster presentations, case reports, and letters to the editor.

PRISMA guidelines supported the search and selection of the studies, and the data of the studies were extracted.

## 5. Conclusions

The medical use of CS is on the rise. Although the literature shows important antioxidant and anti-inflammatory, it is still not possible to establish a treatment criterion since the studies have no standardization regarding the variety and part of the plant that is used, route of administration, and doses. Therefore, we suggest caution in the use of CS in the therapeutic approach of IBD until clinical trials with standardization and a relevant number of patients are performed.

Therefore, we suggest caution in the use of CS in the therapeutic approach of IBD until clinical trials with standardization and a relevant number of patients are performed, and because many side effects can be associated with this plant and its derivatives.

## Figures and Tables

**Table 1:** Main characteristics of the studies included in the review.

Reference	Type and Country of the Study	Patients/Intervention	Outcomes	Side Effects	Main Conclusions
Mbachi et al. 2019 [22]	Population-based cohort study/United States)	39,806 individuals with UC (23–69 y; 26,391 men). Cannabis users: 298; non-users: 39,508	Evaluation of clinical end-points showed lower bowel obstruction (6.4% versus 12.0%), and partial or total colectomy (4.4% versus 9.6%). Cannabis users presented shorter hospital lengths of stay.	Not reported	Cannabis may mitigate some of the well-described complications of UC among hospitalized patients.
Kerlin et al. 2018 [20]	Longitudinal, internet-based cohort study/Israel	1666 Individuals (CD: 1045; UC: 121; 116 women; 503 men) who completed a baseline health survey with updates every 6 m. Cannabis users (recreational or prescription): 114; non-users: 1552. The patients who complete a survey on marijuana were included.	The majority of marijuana users (80.7%) perceived improvement in pain (68%), appetite (49%), anxiety (48%), fatigue (26%), stool frequency (23%), weight gain (20%), and blood in the stool (5%)).	Anxiety, pain, depression, and lower social satisfaction.	Users reported clinical improvement of IBD symptoms, but they reported more anxiety, depression, and pain. Marijuana use may be higher in patients with IBD symptoms not well treated by conventional medical approaches.
Irving et al. 2018 [18]	Multicenter, randomized, double-blind, placebo-controlled study/12 weeks/United Kingdom	60 mild to moderate UC patients refractory to 5-ASA (16 women; 44 men). Placebo group n = 31 (42.8 ± 12.9 y) and treated group (n = 29; 44.8 ± 15.1 y) that received oral hard gelatin capsules with 50 mg CBD-RBE, 2xd, 30 min before morning and evening meals. Patients entered a 2-week dose-escalation period and were required to reach their maximum tolerated dose of up to 250 mg, 2xd/6 weeks.	Remission was observed in both groups at about equal levels. Treated group reported a reduction in the severity of the disease, abdominal pain, and reported feeling better. NO differences were found for stool, bleeding, and levels of IL-2, IL-6, and TNF-α.	Dizziness and somnolence	Treated group showed clinical remission but without statistical significance. Patients treated with *cannabis* reported improvement in the quality of life, showing potential to treated UC.
Naftali et al. 2017 [16]	Double-blind, randomized placebo-controlled trial/8 weeks/Israel	19 patients with active DC (8women; 11men). Placebo group: n = 9 (20–50 y), and treated group: n = 10 (18–75 y) that received oral CBD oil (05 mg/about 0.3 mg/kg) or placebo 2xd.	No clinical improvement of CDAI was observed after oral CBD. Hemoglobin, albumin, CRP, and kidney and liver function tests remained unchanged with the treatment.	Side effects did not differ between the groups.	Patients showed clinical remission without statistical significance, and any other beneficial effect was reported.
Naftali et al. 2013 [15]	Double-blind, randomized, placebo-controlled trial/8 weeks/Israel	21 patients with active CD (9 women; 12men). Placebo group: n = 10 (26–48 y); and treated group: n = 11 (29–63 y). The recommendation was inhalation of cannabis, 2xd, in the form of cigarettes containing 11.5 mg of THC. The cigarettes of the placebo group contained cannabis flowers.	Complete remission (CDAI score <150) was achieved by 5/11 subjects in the cannabis group and 1/10 in the placebo group. A decrease in CDAI score of >100 was observed in 10/11 subjects in the cannabis group and 4/10 in the placebo group. Moreover, it improved appetite and sleep.	No significant differences in side effects (confusion, sleepiness, and nausea) for both groups.	Patients showed clinical remission without statistical significance. However, the administration of inhaled cannabis provided benefits in clinical response and steroid dependence.
Lahat et al. 2012 [14]	Open-label, prospective and single-arm trial/3 months/Israel	13 patients (4 women; 9 men); CD: 11 (28–62 y), UC: 2 (28–31 y) were instructed to use cigarettes with 50 g of dry processed cannabis (inhaled) whenever they observed pain. They were guided to take up to 3 inhalations from the prepared cigarettes each time for 3 months.	After treatment, patients reported improvement of daily activities, decreased pain, improvement of general health perception, patients’ ability to work and to maintain social activities. Patients also presented weight gain.	Not reported	Administration of inhaled cannabis can promote clinical improvement in patients with IBD.
Naftali et al. 2011 [13]	Retrospective observational study/Israel	30 patients (26 men and 4 women; 21–65 y) with CD using cannabis (because of lack of response to conventional therapy) were interviewed. Four patients used recreational cannabis.	Most patients used inhaled cannabis (*joints*) or through water (*bongs*), and all of them reported that the use of cannabis reduced disease activity (Harvey–Bradshaw scale) and the use of other medications.	Not reported	The use of cannabis shows positive effects on CD activity.

THC: Δ9-tetrahydrocannabinol; TNF-α: tumor necrosis factor; IL: interleukin; CBD: cannabidiol; CBD-RBE: CBD-rich botanical extract; CD: Crohn’s disease; CDAI: Crohn’s disease activity index; IBD: inflammatory bowel disease; UC: ulcerative colitis; 5-ASA: 5-aminosalicylic acid; CRP: C reactive protein; d: day; w: week; m: month.

**Table 2:** Some relevant phytocompounds found in *Cannabis sativa* and possible actions.

Phytocompound	CB1	CB2	Others		
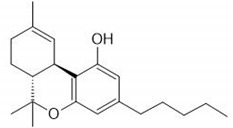 9δ-tetrahydrocannabinol	++	+	Antagonist of 5HT3A: reduction of emesis and pain; Agonist of peroxisome proliferator activated receptor gamma: vasorelaxation of the aorta and superior mesenteric arteries.	References [10,33,53,59,60,61,62]	**CB1 receptor:** *Central, peripheral and enteric nervous system* Reduction of excitatory neurotransmission, causing decreased intestinal motility Reduction of glutamate of the dorsal vagal complex reducing emesis Reduced peripheral inflammatory hypersensitivity and hyperalgesia, reducing pain Reduction of gastric acid production **CB2 receptor:** *Immune tissues (macrophages, neutrophils, epithelial cells, B cells and T cells)* Increases IL-10 levels Produces antinociception Reduction of inflammatory edema
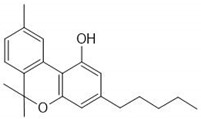 Cannabinol	+	++	Agonist of transient receptor potential cation channel (TRPA1).		
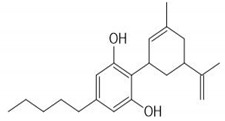 Cannabidiol	+	+	Antagonist of GPR55; Agonist of Adenosine A1A and A2A receptor: inflammation reduction; inhibition of the equilibrative nucleoside transporter.		
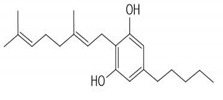 Cannabigerol	+	NR	Agonist of α2 adrenoceptor; Antagonist of 5HT1A; Agonist of TRPA1; Antagonist TRPM8.		
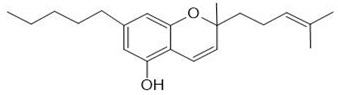 Cannabicrhomene	I	I	Agonist of TRPA1; Agonist of TRPV3; Agonist of TRPV4.		

^++^: Agonist with high affinity; ^+^: Agonist with low affinity; NR: not reported; I: insignificant; CB1: cannabinoid receptor type 1; CB2: cannabinoid receptor type 2; 5HT3A: 5-hydroxytryptamine receptor 3A; GPR55: G-protein coupled receptor 55; 5HT1A: 5-hydroxytryptamine receptor 1A; TRPA1: transient receptor potential cation channel subfamily A member 1; TRPM8: transient receptor potential cation channel subfamily M member 8; TRPV3: transient receptor potential cation channel subfamily V member 3; TRPV4: transient receptor potential cation channel subfamily V member 4.
